# A novel class of TMPRSS2 inhibitors potently block SARS-CoV-2 and MERS-CoV viral entry and protect human epithelial lung cells

**DOI:** 10.1073/pnas.2108728118

**Published:** 2021-10-11

**Authors:** Matthew Mahoney, Vishnu C. Damalanka, Michael A. Tartell, Dong hee Chung, André Luiz Lourenço, Dustin Pwee, Anne E. Mayer Bridwell, Markus Hoffmann, Jorine Voss, Partha Karmakar, Nurit P. Azouz, Andrea M. Klingler, Paul W. Rothlauf, Cassandra E. Thompson, Melody Lee, Lidija Klampfer, Christina L. Stallings, Marc E. Rothenberg, Stefan Pöhlmann, Sean P. J. Whelan, Anthony J. O’Donoghue, Charles S. Craik, James W. Janetka

**Affiliations:** ^a^Department of Biochemistry and Molecular Biophysics, Washington University School of Medicine, Saint Louis, MO 63110;; ^b^ProteXase Therapeutics, Inc., Saint Louis, MO 63108;; ^c^Department of Molecular Microbiology, Washington University School of Medicine, Saint Louis, MO 63110;; ^d^Program in Virology, Harvard Medical School, Boston, MA 02115;; ^e^Department of Pharmaceutical Chemistry, University of California, San Francisco, CA 94158;; ^f^Skaggs School of Pharmacy and Pharmaceutical Sciences, University of California San Diego, La Jolla, CA 92093;; ^g^Infection Biology Unit, German Primate Center, Leibniz Institute for Primate Research, Göttingen 37077, Germany;; ^h^Faculty of Biology and Psychology, Georg-August University Göttingen, Göttingen 37077, Germany;; ^i^Division of Allergy and Immunology, Cincinnati Children’s Hospital Medical Center and Department of Pediatrics, University of Cincinnati College of Medicine, Cincinnati, OH 45229

**Keywords:** antiviral, protease inhibitor, COVID-19, structure-based drug discovery, PS-SCL

## Abstract

The host cell serine protease TMPRSS2 is an attractive therapeutic target for COVID-19 drug discovery. This protease activates the Spike protein of severe acute respiratory syndrome coronavirus 2 (SARS-CoV-2) and of other coronaviruses and is essential for viral spread in the lung. Utilizing rational structure-based drug design (SBDD) coupled to substrate specificity screening of TMPRSS2, we have discovered covalent small-molecule ketobenzothiazole (kbt) TMPRSS2 inhibitors which are structurally distinct from and have significantly improved activity over the existing known inhibitors Camostat and Nafamostat. Lead compound MM3122 (4) has an IC_50_ (half-maximal inhibitory concentration) of 340 pM against recombinant full-length TMPRSS2 protein, an EC_50_ (half-maximal effective concentration) of 430 pM in blocking host cell entry into Calu-3 human lung epithelial cells of a newly developed VSV-SARS-CoV-2 chimeric virus, and an EC_50_ of 74 nM in inhibiting cytopathic effects induced by SARS-CoV-2 virus in Calu-3 cells. Further, MM3122 blocks Middle East respiratory syndrome coronavirus (MERS-CoV) cell entry with an EC_50_ of 870 pM. MM3122 has excellent metabolic stability, safety, and pharmacokinetics in mice, with a half-life of 8.6 h in plasma and 7.5 h in lung tissue, making it suitable for in vivo efficacy evaluation and a promising drug candidate for COVID-19 treatment.

## Significance

MM3122 represents an advanced lead candidate for clinical development as a novel antiviral drug for COVID-19. In addition to being novel drugs, these selective TMRSS2 inhibitors can be used as valuable chemical probes to help elucidate mechanisms of viral pathogenesis. Since TMPRSS2 plays a key role as a viral protein processing protease in the pathogenesis of other coronaviruses (SARS-CoV, MERS-CoV) as well as influenza viruses, MM3122 and this class of TMPRSS2 inhibitors hold much promise as new drugs to not only treat SARS-CoV-2 infections but also potentially represent broad-spectrum antivirals.

Severe acute respiratory syndrome coronavirus 2 (SARS-CoV-2) is the newly emerged, highly transmissible coronavirus responsible for the ongoing COVID-19 pandemic, which is associated with 136 million cases and almost 3 million deaths worldwide as of April 12, 2021 (https://coronavirus.jhu.edu/map.html). While three vaccines have recently been approved by the FDA, there are still no clinically approved small-molecule drugs available for the treatment of this disease except Remdesivir, and the effectiveness of the vaccines against immune escape variants might be reduced. Multiple therapeutic strategies have been proposed (1−2), including both viral and host proteins, but none have yet been fully validated for clinical application. One class of protein targets which have shown promising results are proteolytic enzymes including the viral proteases (1, 3–5), Papain-Like Protease (PLpro) and the 3C-like or “Main Protease” (3CL or MPro), and several host proteases involved in viral entry, replication, and effects on the immune system creating the life-threatening symptoms of COVID-19 infection (4–6). The latter include various members of the cathepsin family of cysteine proteases, including cathepsin L, furin, and the serine proteases factor Xa, plasmin, elastase, tryptase, TMPRSS2, and TMPRSS4.

Coronavirus (SARS CoV-2, SARS-CoV, and Middle East respiratory syndrome coronavirus [MERS]) entry is mediated by the viral spike protein, which must be cleaved by host proteases in order to trigger membrane fusion and entry into the host cell after binding to the host cell receptor Angiotensin Converting Enzyme-2 (ACE2) (7–10). This is mediated by initial cleavage at the S1/S2 junction of spike, which is thought to occur during processing in the producer cell, followed by cleavage at the S2′ site either by serine proteases at the cell surface or by cathepsin proteases in the late endosome or endolysosome (9, 10). Whether serine or cathepsin proteases are used for S2′ cleavage is cell type dependent. While entry into Calu-3 (human lung epithelial) or HAE (primarily human airway epithelial) cells is cathepsin independent, entry into Vero cells (African green monkey kidney epithelial), which do not express the required serine proteases, depends exclusively on cathepsins (7, 9–11).

TMPRSS2 (12) is a type II transmembrane serine proteases (TTSP) (13) that has been shown to be crucial for host cell viral entry and spread of SARS-CoV-2 (7, 8, 14–16), as well as SARS-CoV (17, 18), MERS-CoV (19), and influenza A viruses (20–27). The Spike protein requires proteolytic processing/priming by TMPRSS2 to mediate entry into lung cells; thus, small-molecule inhibitors of this target offer much promise as new therapeutics for COVID-19 and other coronavirus diseases (7, 8). TMPRSS2 expression levels dictate the entry route used by SARS-CoV-2 to enter cells, as reported recently (28). In cells that express little or no TMPRSS2, cell entry occurs via the endosomal pathway, and cleavage of spike protein is performed by cathepsin L. It has been demonstrated that the TMPRSS2-expressing lung epithelial Calu-3 cells are highly permissive to SARS-CoV-2 infection. The irreversible serine protease inhibitors Camostat (7) and Nafamostat (29) are effective at preventing host cell entry and replication of SARS-CoV-2 in Calu-3 cells through a TMPRSS2-dependent mechanism (14, 15).

Herein, we report on the discovery of a class of substrate-based ketobenzothiazole (kbt) inhibitors of TMPRSS2 with potent antiviral activity against SARS-CoV-2 which are significantly improved over Camostat and Nafamostat. Several compounds were found to be strong inhibitors of viral entry and replication, with EC_50_ (half-maximal effective concentration) values exceeding the potency of Camostat and Nafamostat and without cytotoxicity. Newly developed compound MM3122 (**4**) has excellent pharmacokinetics (PK) and safety in mice and is thus a promising lead candidate drug for COVID-19 treatment.

## Results and Discussion

### Hit Identification of TMPRSS2 Inhibitors.

It is well established that several TTSPs including TMPRSS2 play a role not only in infectious diseases (12) but also in cancer progression and metastasis (30–32) which is thought to be mainly through their ability to activate hepatocyte growth factor (HGF), the sole ligand for MET receptor tyrosine kinase. This is accomplished via proteolytic processing of the inactive single-chain precursor pro-HGF to a two-chain active form. TMPRSS2 shares pro-HGF as a protein substrate with the other HGF-activating serine proteases, HGFA (HGF-Activator), hepsin, and matriptase (33, 34). Like TMPRSS2, other TTSPs such as matriptase and hepsin have a canonical serine protease domain residing as the C-terminal domain of the protein that is anchored to the cell membrane by an N-terminal type II signal peptide, thereby presenting their enzymatic activity outside the cell. We have previously reported on the discovery and anticancer properties of peptidomimetic ketothiazole (kt) and kbt inhibitors of HGFA, matriptase, and hepsin (35–39) named synthetic HGF Activation Inhibitors or sHAIs. Since TMPRSS2 has an overlapping endogenous substrate specificity profile with HGF-activating proteases, we postulated that our substrate-based sHAIs would also inhibit TMPRSS2.

#### Antiviral activity of sHAIs (VSV/SARS-CoV-2 spike protein pseudotypes).

**Fig. 1.**
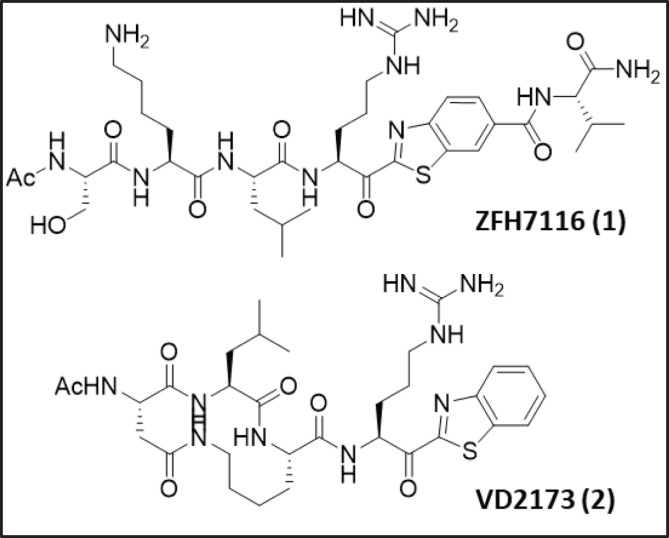
Structures of the initial of TMPRSS2 inhibitors discovered by screening existing HGFA, matriptase, and hepsin serine protease inhibitors.

**Fig. 2.**
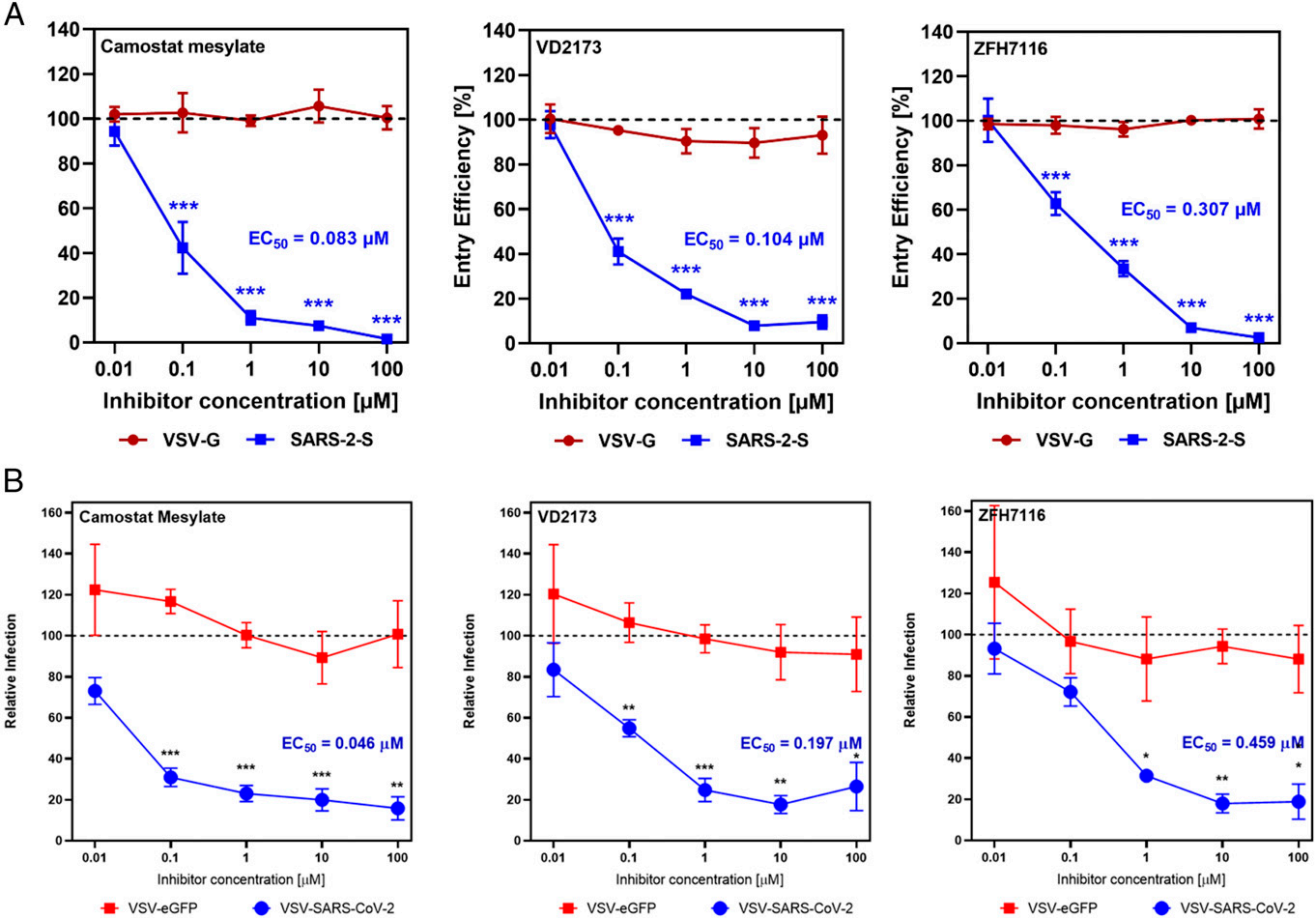
Inhibition of SARS-CoV-2 cell entry into Calu-3 lung epithelial cells by ZFH7116 (**1**) and VD2173 (**2**) using (*A*) VSV-SARS-CoV-2-Spike protein Pseudotype virus and (*B*) VSV-SARS-CoV-2-Spike protein Chimeric virus. EC_50_s are calculated from an average of three separate experiments. Statistics compare relative infection at each concentration (*P < 0.05, **P < 0.01, ***P < 0.001, Student's *t* test). Camostat was used as a positive control.

Based on our premise that sHAIs would inhibit TMPRSS2, we first selected the two sHAI lead compounds, ZFH7116 (**1**) (37, 40) and VD2173 (**2**) (Fig. 1), for initial antiviral testing and confirmed that they potently inhibit TMPRSS2-dependent host cell viral entry driven by the spike protein of SARS-CoV-2 (SARS-2 S; Fig. 2*A*) into Calu-3 lung epithelial cells having an EC_50_ of 307 and 104 nM, respectively. Marked inhibition of SARS-2 S−driven entry was also detected for the irreversible inhibitor Camostat, in line with published data (7, 29). It is noteworthy that neither compound showed activity in TMPRSS2-negative Vero cells or modulated entry into Calu-3 cells by pseudotypes bearing the VSV glycoprotein (VSVpp VSV-G) (*SI Appendix*, Fig. S6), suggesting that the inhibition of SARS-CoV-2 spike-driven entry into Calu-3 cells was due to blockade of TMPRSS2.

#### Antiviral activity of sHAIs (VSV/SARS-CoV-2 spike protein chimeras).

Using a replication-competent, chimeric VSV expressing the SARS-CoV-2 Spike [shown to mimic the Spike-dependent entry of authentic SARS-CoV-2 (41)], we also demonstrated that **1** and **2** block viral entry into Calu-3 cells in a dose-dependent manner. Both drugs displayed EC_50_ values of 459 and 197 nM against the chimeric virus VSV-SARS-CoV-2 (Fig. 2*B*) compared to the pseudotyped virus (Fig. 2*A*), while showing no activity against VSV-eGFP (VSV G−dependent entry), or against either virus in Vero cells (*SI Appendix*, Fig. S7), which are TMPRSS2 negative. This confirms our initial result and allows for the establishment of a system for screening antiviral activity of these inhibitors using VSV-SARS-CoV-2.

#### Inhibition of cell-bound TMPRS2 enzymatic activity by sHAIs.

We tested the inhibitory activity of **1** and **2** on TMPRSS2 proteolytic activity in a cell-based enzyme-based fluorogenic assay, by overexpression of TMPRSS2 in a human cell line, HEK-293T, commonly used for experimentation because of its high transfectability. As previously demonstrated, expression of TMPRSS2 can be accurately measured (42, 43) using a fluorogenic peptide reporter substrate, Boc-QAR-AMC, in cell cultures. Compound **1** inhibited cell-based TMPRSS2 enzyme activity in a concentration-dependent manner between 10 μM and 10 nM, with an IC_50_ (half-maximal inhibitory concentration) of 314 nM (*SI Appendix*, Fig. S4). Compound **2** was a more potent inhibitor of TMPRSS2 proteolytic activity, with an IC_50_ of 57 nM. These data confirm that both **1** and **2** mediate their function by potently inhibiting TMPRSS2.

#### Selectivity data of TMPRSS2 inhibitors in a panel of proteases.

We profiled **1** and **2** for their selectivity against a panel of 43 serine and cysteine proteases (*SI Appendix*, Tables S1 and S2) for comparison to that of Camostat and Nafamostat protease selectivity data which have been published (44). We found **1** was a potent inhibitor of matriptase-2, plasma kallikrein, proteinase K, trypsin, tryptase b2, and G1 but also was a moderate inhibitor of factor Xa, factor XIIa, and kallikreins 5 and 14, as well as cysteine protease cathepsin S, while showing some activity against cathepsin L as well. There was overlap of **1** with the selectivity profiles of Camostat, except they did not show any activity against the cathepsins. Furthermore, Camostat more potently inhibited factor XIIa, plasma kallikrein, matriptase-2, and plasmin, while also having increased activity for trypsin, the tryptases, and urokinase. Cyclic peptide 2 also more potently inhibited urokinase and factor XIIa relative to **1** but not cathepsin S or L, and instead was a moderate inhibitor of cathepsin B. The ramifications of these different selectivity profiles may explain the varied activity in the VSV-pseudotype and chimeric viral entry results most notably the urokinase potency for which **1** was significantly weaker.

### Lead Identification of TMPRSS2 Inhibitors.

**Fig. 3.**
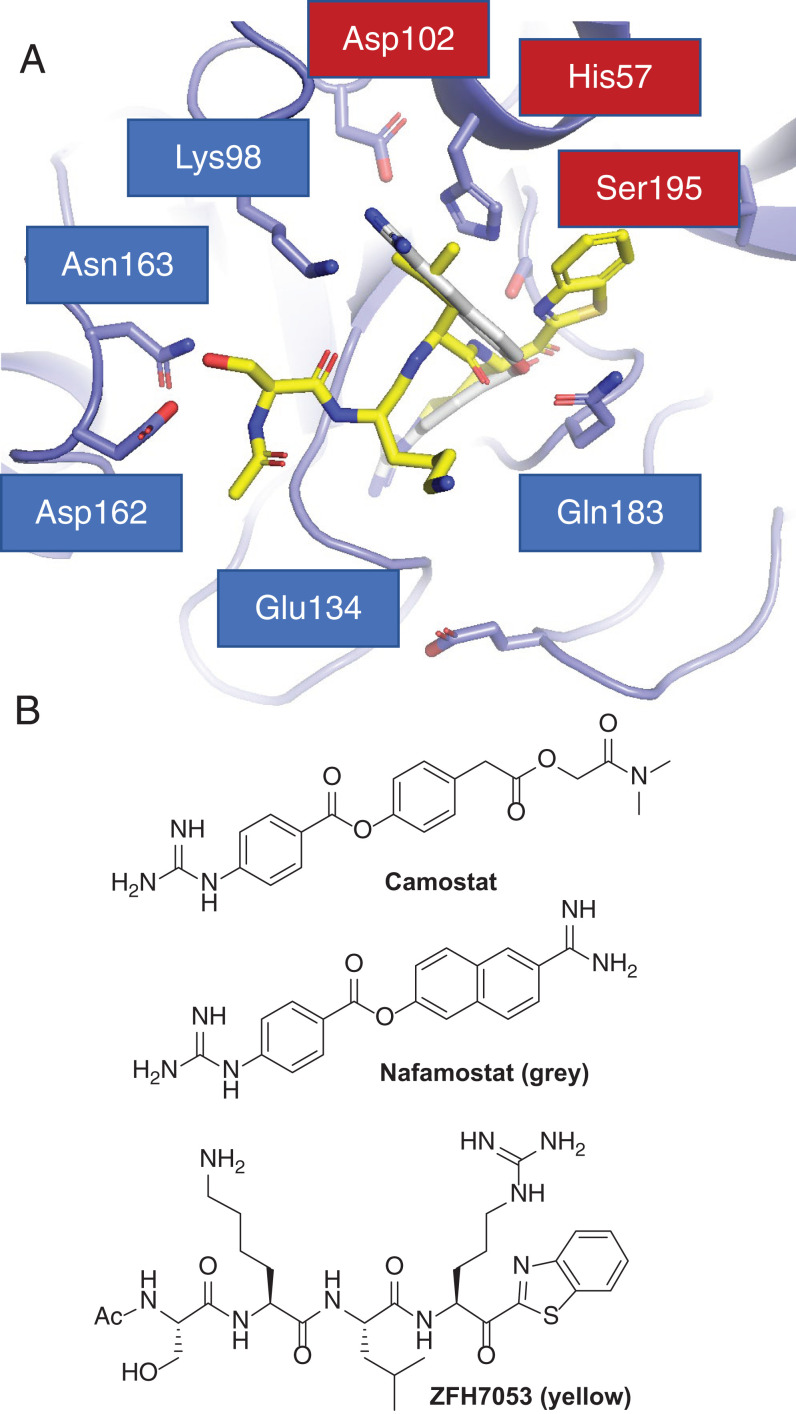
(*A*) Molecular docking model of compound **3** (yellow) and Nafamostat (gray) bound to a homology model of TMPRSS2 using Glides/Schrödinger. (*B*) Structures of compound **3**, Camostat, and Nafamostat.

We published (38) that Camostat and Nafamostat (Fig. 3*B*) are inhibitors of matriptase and hepsin, and analog Nafamostat is the most active published inhibitor of SARS-CoV-2 cell entry (8). While we previously pursued this chemical series as HGFA, matriptase, and hepsin inhibitors (38), the best series we have developed are small peptide-based molecules like **1**,** 2**, and **3** (Ac-SKLR-kbt; Fig. 3*B*) which exhibit low nanomolar to picomolar IC_50_s. These compounds contain a serine-trapping kbt warhead (35–37, 39) which reacts covalently with the protease, but, importantly, in a reversible manner, unlike Camostat or Nafamostat, in which the inhibition is irreversible. Therefore, we pursued the kbt class of inhibitors for lead identification studies toward more potent and selective TMPRSS2 inhibitors.

#### Rational design of TMPRSS2-selective kbt inhibitors.

We have employed X-ray cocrystal structure data in the rational design of optimized HGFA, matriptase, and hepsin inhibitors with increased potency and selectivity (35–38). Shown in Fig. 3, here we used a homology model of human TMPRSS2 (45) to computationally model our existing inhibitors (using Glide in Schrödinger) where we docked compound **3** (yellow; Fig. 3*A*) and Nafamostat (gray). For Nafamostat, the naphthyl portion extends deep into the S2 pocket, with the benzguanidine in the S1 pocket binding to the conserved S1 Asp189. The P4 Ser of **3** makes two H bonds to Asp162 and Asn163 outside the S4 pocket, and the Ser backbone carbonyl is also within H-bonding distance of the P2 Lys98. The P3 Lys is near the S4 Glu134 making an electrostatic interaction, while the P2 Leu resides in the S2 pocket, and the benzothiazole fills the S1′ area. The reactive ester of Nafamostat and ketone of the kbt are adjacent to the Ser195−His57−Asp102 catalytic triad. Our model of **3** and Nafamostat bound to TMPRSS2 (Fig. 3*A*) shows a nice fit to the S1 through S4 and S1′ pockets of TMPRSS2. For **3**, it appears that the P4 Ser is making a dual H bond to the Asp162 and Asn163 residues in TMPRSS2. This area is occupied by a Gln in both hepsin and matriptase but by an Asp in HGFA, and, interestingly, the additional Asn163 residue is unique to TMPRSS2. This suggests that a free amine in the P4 could provide selectivity over matriptase and hepsin. The structure also shows that Lys87 resides in the S2 pocket, suggesting a P2 Glu or Asp might be ideal for an inhibitor of TMPRSS2 and lead to potential selectivity over HGFA (P2 Ser), matriptase (P2 Phe), and hepsin (P2 Asn). This Lys87 is also near the P4 Ser side chain, indicating an Asp or Glu in this position could potentially make a third electrostatic interaction with Lys87. Further, the S2 pocket is large and appears able to accommodate larger side chains such as Phe, Tyr, or Trp.

#### PS-SCL protease substrate specificity profiling of TMPRSS2.

**Fig. 4.**
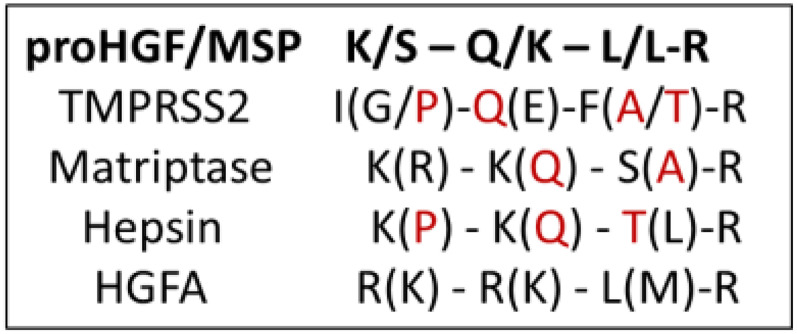
Combined PS-SCL for substrate specificity of TMPRSS2, matriptase, hepsin, and HGFA. Using protease substrate terminology, the cleavage (hydrolysis) site for protease protein substrates is between the N-terminal P1 and P1′ positions of the peptide (P) substrate, while S1 and S1′ refer to the subsite (S) 1 and 1′ of the protease where the P1 and P1′ amino acid side chain binds (46). Red color designates overlap of TMPRSS2 amino acid specificity with other proteases. Single letter designations for amino acids are shown.

To augment our rational design of more potent and selective TMPRSS2 inhibitors, we analyzed existing data on the peptide substrate preferences of TMPRSS2, HGFA, matriptase, and hepsin. When comparing the positional scanning of substrate combinatorial libraries (PS-SCL) data of TMPRSS2 (Fig. 4) (30) with that of matriptase (47), hepsin (48), and HGFA (36), it became apparent that there was significant overlap in their preferences for substrates. These data reveal that TMPRSS2 is tolerant of many different P2 side chains but prefers Phe and Ala/Thr like matriptase (Fig. 4), which can also tolerate both large and small groups (prefers Ser/Ala), but hepsin and HGFA both prefer Leu. For P3, TMPRSS2 prefers Gln/Glu and Met, whereas Lys/Gln is preferred for hepsin and matriptase, and Lys/Arg is preferred for HGFA (Fig. 4). The clearest distinction is in the P4 position, where HGFA, matriptase, and hepsin all prefer basic residues like Lys/Arg, while TMPRSS2 prefers Ile/Gly and Pro, which is a shared attribute with hepsin.

#### Multiplex substrate profiling by mass spectrometry profiling of TMPRSS2 for extended substrate sequence specificity.

**Fig. 5.**
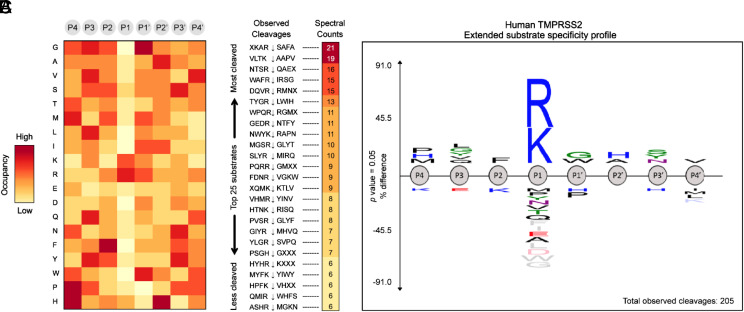
(*A*) Heatmap displaying the overall amino acid frequencies in each of the P4−P4′ positions of a positive set of peptides cleaved by human TMPRSS2. Positive enrichment is observed as red, and zero was set to yellow. (*B*) Total spectral counts determined by LC-MS/MS highlight peptide sequences as high turnover rates substrates for TMPRSS2. (*C*) IceLogo depicting the extended substrate specificity of human TMPRSS2 based on 205 cleavage events detected through MSP-MS analysis.

To strengthen our compound design, we obtained further information on the extended substrate specificity of TMPRSS2 using multiplex substrate profiling by mass spectrometry (MSP-MS) (49). In the MSP-MS assay, a physiochemically diverse library of 228 tetra decapeptides was incubated for several hours with human recombinant TMPRSS2 in activity buffer. At different time points (15, 60, and 240 min), aliquots of the reaction mixture were extracted, quenched with 8 M guanidium HCl, and analyzed by tandem MS to allow monitoring of protease-generated cleaved products. As a TTSP with a trypsin fold, TMPRSS2 is known for its high affinity toward substrates containing an arginine residue in the P1 position (30). This prominent P1 specificity was confirmed by our MSP-MS analysis, which showed a nearly exclusive occupancy of the P1 position by either Arg or Lys on TMPRSS2-generated cleaved products (Fig. 5*A*). Peptide sequencing by LC-MS/MS enabled the identification of the 25 most preferred substrates for TMPRSS2 in our peptide library (Fig. 5*B*). An IceLogo frequency plot (Fig. 5*C*) displays the extended substrate specificity profile of TMPRSS2 at pH 7.5, which reveals the preference for hydrophobic amino acids in both P2 and P1′ positions, which flank the cleavage site. The analysis indicates a preferred cleavage of N-terminal tetrapeptides to be PLFR with other P4 amino acids H and M and P3 amino acids G, Y, V, and Q but only F at P2, which is striking. Largely, these data recapitulate what was elucidated in the PS-SCL study (Fig. 4). Based on careful analysis of the computational modeling work with these and the PS-SCL results, we selected additional existing compounds in our library of HGFA/matriptase hepsin inhibitors for testing and synthesized (*SI Appendix*, Schemes S1–S3) four analogs specifically designed for TMPRSS2: Ac-GQFR-kbt (**4**), Ac-PQFR-kbt (**5**), Ac-QFR-kbt (**6**), and Ac-IQFR-kbt (**7**).

#### Inhibition of VSV-SARS-CoV-2 viral entry.

Analogs based on the initial hit compounds **1** and **2** along with the substrate specificity data were Ac-SKLR kbt (**3**), Ac-WFR-kbt (**8**), Ac-SKFR-kt (**9**), Ac-KQFR-kt (**10**), Ac-SQLR-kt (**11**), Ac-FLFR-kbt (**12**), Ac-dWFR-kbt (**13**), dWFR-kbt (**14**), dWFR-kbt-CO_2_H (**15**), Ac-WLFR-kbt (**16**), Ac-KQLR-kbt (**17**), Ac-LLR-kt (**18**), Cyclo(DMK)R-kbt (**19**), Cyclo(DQK)R-kbt (**20**), and Cyclo(allylGLY)R-kbt (**21**). Many of these compounds contain a Phe (F) residue in the P2 position as predicted from the PS-SCL study, while others contain a Gln (Q) at the P3 position. When tested in the VSV pseudotype assay, all displayed improved activity relative to **1** (Table 1), where the best compound was **13**. Compound **13** is a tripeptide containing an unnatural amino acid D-Trp in the P3 position and Phe in the P2 position, predicted from PS-SCL and MSP-MS analysis to be preferred. Compound **17,** which contains a P3 Gln also predicted from PS-SCL, shows the next best activity, with an EC_50_ of 78 nM. Furthermore, **16**, also having a P2 Phe, showed an excellent potency of 150 nM. Corresponding cycloamide analogs of **2**, **19**, and **20** retained good activity (EC_50_ of 119 and 138 nM) for inhibition of viral entry of the VSV pseudotype, but allyl phenyl ether analog **21** showed a significant decrease in activity (EC_50_ 565 nM).

**Table 1.** Structures and inhibition data of compounds for protease enzyme activity and VSV pseudotype and chimera SARS-CoV-2 viral entry into human Calu-3 lung epithelial cells

Name/structure	TMPRSS2 IC_50_ (nM)	HGFA IC_50_ (nM)	Matriptase IC_50_ (nM)	Hepsin IC_50_ (nM)	Thrombin IC_50_ (nM)	Factor Xa IC_50_ (nM)	VSV-SARS-CoV-2 Calu-3 EC_50_ (nM)	VSV-SARS-CoV-2 Chimera Calu-3 EC_50_ (nM)	SARS-CoV-2 Wild-type Calu-3 IC_50_ (nM)
Remdesivir	NA	NA	NA	NA	NA	NA	ND	ND	1,271
Camostat	1.5	>20,000	7.0	7.0	>20,000	>20,000	83	21	ND
Nafamostat	0.14	158	0.05	0.9	5,020	4,570	ND	ND	220*
Ac-SKLR-kbt-V (1)	74	23	14	1.0	7530	514	307	489	ND
Cyclo(DLK)R-kbt (2)	2.6	8520	2.6	19	8140	2,050	104	197	660
Cyclo(DMK)R-kbt (19)	19	3240	1.0	5.9	18,500	1,390	119	ND	ND
Cyclo(aGLY)R-kbt (21)	197	>20,000	14	20	>20,000	8,810	565	ND	ND
Cyclo(DQK)R-kbt (20)	ND	16,100	7.7	22	>20,000	>20,000	138	ND	ND
Ac-WFR-kbt (8)	9.4	329	5.7	7.6	>20,000	22	ND	1,377	ND
Ac-SKFR-kt (9)	7.9	114	6.1	17	>20,000	1,060	ND	1,157	ND
Ac-KQFR-kt (10)	29	116	1.4	1.2	>20,000	158	ND	262	ND
Ac-SQLR-kt (11)	16	364	18	0.68	ND	ND	ND	1,320	ND
Ac-LLR-kt (18)	54	506	56	4.6	ND	ND	349	1,838	ND
Ac-SKLR-kbt (3)	39	66	6.1	0.32	>20,000	3,800	ND	4,272	ND
Ac-FLFR-kbt (12)	3.0	228	7.2	2.9	>20,000	26	ND	101	ND
Ac-dWFR-kbt (13)	1.1	27	2.6	1.1	3,700	98	32	ND	ND
dWFR-kbt (14)	39	ND	ND	ND	ND	ND	ND	357	ND
dWFR-kbt-CO_2_H (15)	42	ND	ND	ND	ND	ND	ND	105	ND
Ac-WLFR-kbt (16)	6.3	266	12	0.79	>20,000	2.0	150	ND	ND
Ac-KQLR-kbt (17)	ND	60	1.1	0.17	>20,000	258	78	572	ND
Ac-IQFR-kbt (7)	0.25	30	0.92	0.14	>20,000	792	ND	3.6	102
Ac-QFR-kbt (6)	0.31	14	0.13	0.08	>20,000	1.4	ND	0.53	105
Ac-PQFR-kbt (5)	0.28	75	0.32	0.13	>20,000	199	ND	0.86	52
Ac-GQFR-kbt (4)	0.34	32	0.31	0.19	>20,000	700	ND	0.43	74

ND, not determined; NA, not applicable.

* Here, *n* = 1.

**Fig. 6.**
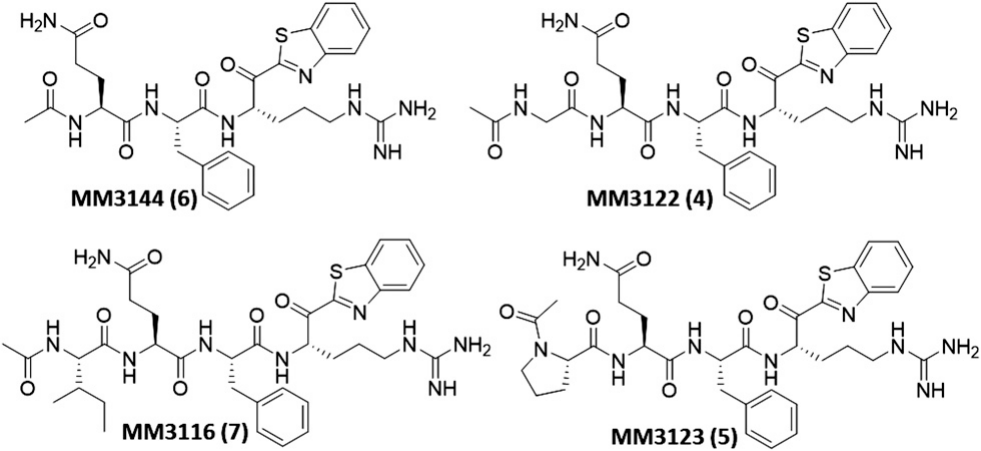
Structures of rationally designed covalent reversible kbt inhibitors (**4** through **7**) of TMPRSS2.

The remaining compounds were tested in the VSV-SARS-CoV-2 chimeric assay. Excitingly, the most promising results (Table 1) were derived from our new rationally designed TMPRSS2 inhibitors, **4** through** 7**. These compounds were designed by incorporating the preferred side chains (as determined from PS-SCL data above) at all the P1 through P4 positions of the inhibitor. The set of existing compounds all showed good potency, with the best, **12**, showing an EC_50_ of 101 nM in blocking viral entry of VSV-SARS-CoV-2 chimeras into Calu-3 cells, sixfold more active than the second-best analog in the pseudotype assay, **17**. Excitingly, three of the new rationally designed analogs, **4** through **6**, displayed significantly increased potency (Fig. 6), with subnanomolar EC_50_s, and the fourth new compound, **7**, is still extremely potent, with an EC_50_ of 3.6 nM. This not only constitutes a 230-fold increase in activity for **4** (EC_50_ 0.43 nM) relative to the previous best compound, **12**, but also an 80-fold improvement over Camostat. Of the other compounds, similar potency compared to **12** was seen from tripeptide **15** (EC_50_ 105 nM) but less so from **14**, analogs of **13**, which was the best compound tested in the pseudotype assay. Also showing excellent potency is **10**, with an EC_50_ of 262 nM.

#### Inhibition of VSV-MERS viral entry.

To demonstrate broad coronavirus activity, we tested Camostat, **2**, and our best new compounds, **4** through **7** (Fig. 6), based on the VSV chimera results for their activity in blocking host cell entry of a MERS-CoV chimeric virus. Shown in Table 2, all compounds inhibited MERS-CoV viral entry with nearly equivalent potency as compared to SARS-CoV-2 (Table 1). Although the TMPRSS2 dependence of MERS-CoV has been demonstrated previously, this provides strong evidence that our compounds have broad-spectrum potential against other coronaviruses in the clinic. To confirm that this activity is indeed TMPRSS2 related, we next set out to produce recombinant TMPRSS2 protein for testing our compounds for their enzyme inhibitory activity.

**Table 2.** Activity of lead compounds against VSV pseudotype MERS viral entry into human Calu-3 lung epithelial cells

Name/structure	MERS VSV pseudotype Calu-3 EC_50_ (nM)	Mouse plasma stability t1/2 (min)	Human plasma stability t1/2 (min)
Camostat	5.0	ND	ND
**2** (VD2173)	273	222	>289
**7** (MM3116)	2.3	154	>289
**6** (MM3144)	0.55	>289	>289
**5** (MM3123)	2.9	>289	>289
**4** (MM3122)	0.87	>289	>289

Also shown is the plasma stability of selected compounds in both mouse and human plasma.

#### Expression and purification of recombinant TMPRSS2 (protease domain).

Recombinantly expressed TMPRSS2 protease domain (*SI Appendix*, Fig. S8*A*) was purified via Ni-nitrilotriacetate (NTA) column (*SI Appendix*, Fig. S8*C*), then verified by sodium dodecyl sulfate polyacrylamide gel electrophoresis (SDS/PAGE) and Western blot (*SI Appendix*, Fig. S8 *D* and *E*), showing no presence of other proteins. Catalytic activity was tested by combining 150 nM of purified TMPRSS2 protease domain with (MCA)-K-KARSAFA-K-(DnP), an 8-mer peptide fluorescence resonance energy transfer (FRET) substrate that was synthesized based on the peptide that was most efficiently preferred by TMPRSS2 from our MSP-MS analysis (Fig. 5). Michaelis−Menten kinetics showed a K_m_ of 0.6 ± 0.05 μM (*SI Appendix*, Fig. S8*B*). Relative to the full-length TMPRSS2 (see below), the k_cat_ (catalytic rate constant) value achieved for the protease domain (0.07/s) was 1,000-fold lower than that observed for the construct bearing the LDLR class A domain and scavenger receptor cysteine-rich (SRCR) modules N-terminal to the catalytic domain (k_cat_ = 94.54/s). Further investigations are necessary to understand these differences and the interactions among the catalytic domain, the LDLR, and the SRCR domains that impact the extended specificity and, potentially, the catalytic activity of TMPRSS2.

#### Recombinant TMPRSS2 (full-length) enzyme assay.

Using active recombinant full-length TMPRSS2 as described above, along with Boc-QAR-AMC as a fluorogenic substrate (44), we found the K_m_ (Michaelis constant) was 85.6 μM using an enzyme concentration of 3 nM (*SI Appendix*, Fig. S2). We used this substrate to test all inhibitors for inhibition of TMPRSS2 proteolytic enzyme activity in a standard kinetic assay using Nafamostat and Camostat as controls, where we determined the compound IC_50_s over the period of 1 h, following 30-min compound incubation with enzyme. The IC_50_ values for Camostat and Nafamostat are 1.5 and 0.14 nM, which is similar but slightly improved compared to values previously reported of 6.2 and 0.27 nM, respectively (44). We found the IC_50_ data we generated for TMPRSS2 inhibition closely correlated with the VSV pseudotype and chimera assay data, with some exceptions. Multiple compounds were significantly more potent than Camostat, and several were equipotent to Nafamostat (Table 1). The new rationally designed lead compounds **4** through **7** (Fig. 6) all displayed exquisitely potent subnanomolar IC_50_ values of which **7** was the best, with an IC_50_ of 250 pM, similar to that of Nafamostat. It should be noted, however, that Nafamostat is an irreversible inhibitor, while the kbt class of inhibitors are reversible, so it is difficult to directly compare IC_50_ values between the two series having different mechanisms of inhibition. While the initial hit compound **1** still shows good potency against TMPRSS2 (IC_50_ of 74 nM), it was significantly weaker when compared to the rationally designed TMPRSS2 inhibitors, which was expected. However, the other initial hit compound **2** is a much more potent inhibitor of TMPRSS2 activity than **1**, with an IC_50_ of 2.6 nM, only about 10-fold less active than **4** through **7**. It is noteworthy that the P3 to P1 tripeptide **6** without the P4 side chain was almost as active as any of the tetrapeptides **4**, **5**, or **7** which have a P4 residue. However, this compound **6** and the other tripeptides, including **8** and **13**, suffer from high inhibition of the plasma protease Factor Xa which is undesirable for further drug discovery due to potential bleeding side effects in patients.

The least active compound was the aryl ether cyclic peptide **21**, with an IC_50_ of 197 nM, which is, however, still respectable. It is presumed the larger, more constrained aryl ether ring system is not an ideal fit for binding to the TMPRSS2 active site through bridging the S2-S4 pockets. It is important to note that, while TMPRSS2 activity is potent in all inhibitors, most compounds tested are all still relatively active against the other proteases HGFA, matriptase, and hepsin.

The most selective TMPRSS2 analogs are **16**, with an IC_50_ of 6.3 nM, being 40-fold more active than HGFA, twofold over matriptase, and **12**, which is 60-fold more selective over HGFA and twofold against matriptase. As with other compounds in this series of peptidyl kbt inhibitors, in our experience, we have found that deriving selectivity over hepsin is challenging, but one of our best examples turns out to be initial hit **2**, which is fourfold more active against TMPRSS2 relative to hepsin and almost 10-fold more active against matriptase. The consequences of inhibiting these other serine proteases, especially the other HGF-activating proteases, in the COVID-19 scenario are unclear at this time but may be beneficial for treatment rather than detrimental.

There are clear structure−activity relationships (SAR) from the 21 compounds tested. For example, it appears that TMPRSS2 prefers large groups extending beyond the kbt S1′ C-terminal portion of the inhibitor, since **1** has increased activity relative to unsubstituted kbt analog **3**. Strengthening this hypothesis, the compounds with the smaller kt warhead in the P1′ position like seen in **9** through **11** also lose potency relative to analogs with larger kbt. Also, the P3 position is important for activity where it seems basic groups like Lys (K) are undesired (**9**, **3**, **1**), which seems also true for the P4 position as seen with compound **17**. Finally, as predicted from modeling and the PS-SCL data, Phe is clearly preferred in the P2 position. Therefore, it is likely that diverse new analogs can be pursued with other aromatic side chains like Trp and Tyr, which will also produce inhibitors with exquisite TMPRSS2 activity and potentially more selectivity.

Given that the homology model of TMPRSS2 used in this study was exclusive to the catalytic domain of TMPRSS2, both catalytic domain and full length TMPRSS2 were tested against candidate compounds. The inhibition IC_50_ values against the recombinant protease domain (*SI Appendix*, Fig. S1) versus full-length TMPRSS2 show strikingly different values, with estimated IC_50_s being over 100 μM compared to picomolar to double-digit nanomolar IC_50_ values for the commercial full-length TMPRSS2 (Table 1 and *SI Appendix*, Fig. S3). It is not clear, from our current studies, what the direct cause of this noticeable difference is, but we suspect that the protein domains/modules of TMPRSS2 which are N-terminal to the catalytic domain play an important role in ligand interactions and subsequent catalysis.

#### Antiviral activity of lead compounds (wild-type SARS-CoV-2).

**Fig. 7.**
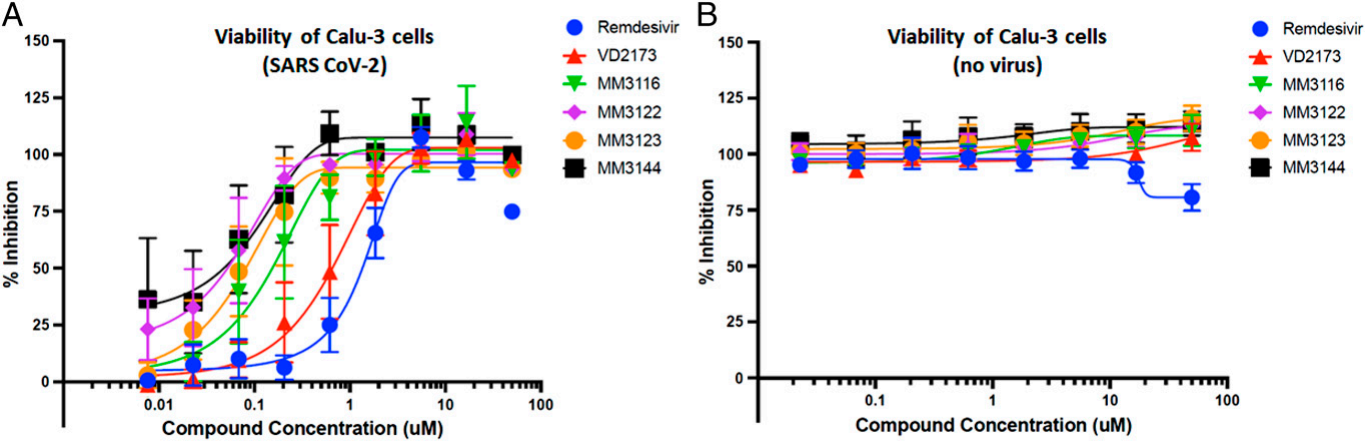
(*A*) Inhibition of the cytopathic effects (viral toxicity) of wild-type SARS-CoV-2 Calu-3 by lead TMPRSS2 inhibitors using the CellTiter-Glo (Promega) assay. (*B*) Viability of Calu-3 cells with compounds in the absence of virus (compound toxicity).

After confirming and quantitating TMPRSS2 enzyme inhibition of all compounds against recombinant protein and their selectivity over HGFA, matriptase, hepsin, thrombin, and Factor Xa, we assessed the activity of our most promising lead compounds **2** and **4** through **7** in inhibiting the cytopathic effects of wild-type SARS-CoV-2 by employing a CellTiter-Glo (Promega) cellular viability assay with lung epithelial Calu-3 cells (50). We used Remdesivir and Nafamostat (*n* = 1) as positive controls. While all compounds showed excellent activity in this assay (Fig. 7 and Table 1), the most potent compounds were the rationally designed TMPRSS2 inhibitors **4** and **5** (Fig. 6), with EC_50_s of 74 and 52 nM, respectively, which is >20-fold improved over Remdesivir and also significantly more active than Nafamostat (note that this is based on *n* = 1). Importantly, all five kbt inhibitors showed no signs of toxicity to Calu-3 cells (up to 50 μM, which is nearly 1,000 times the IC_50_ for **5**), while Remdesivir and Nafamostat displayed toxicity at the highest concentration tested (50 μM).

#### PK and metabolic stability of VD2173 (2) and MM3122 (4).

**Fig. 8.**
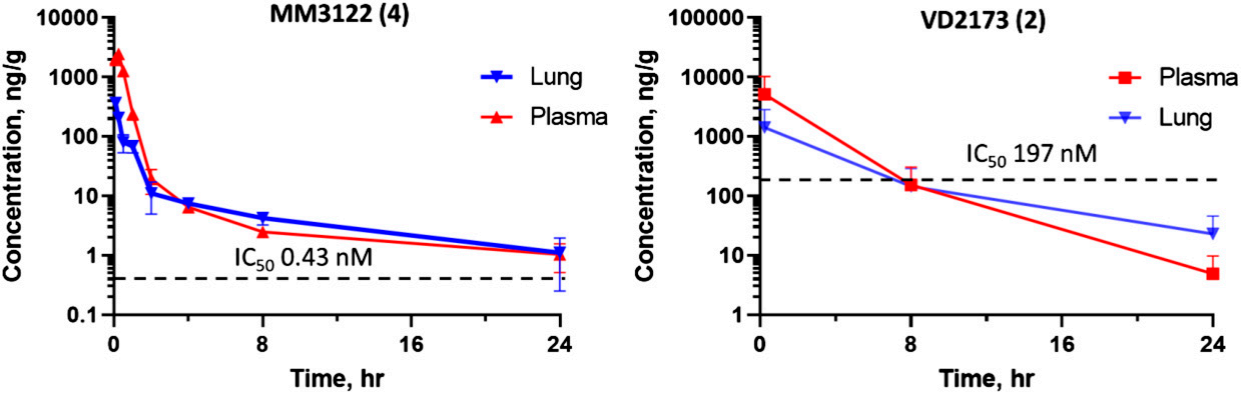
Mouse PK of MM3122 (**4**) and VD2173 (**2**) after IP dosing at 16.7 mg/kg. Compound concentrations over time in plasma and lung tissue are shown relative to the IC_50_ of the compounds in the SARS-CoV-2 VSV hybrid cell entry assay. MM3122 (**4**) has a half-life (t1/2) of 8.6 h in plasma and 7.5 h in the lung, and 2.5 h and 4.2 h, respectively for VD2173 (**2**). Dashed lines indicate the IC_50_ of compounds in the VSVSARS-CoV-2 cell entry assay.

Compound **2** (VD2173) and lead compounds **4** through **7** were tested for their in vitro stability in mouse and human blood plasma (Table 2). All compounds have excellent stability in both mouse and human plasma, with >289 min half-life, except for **7**, which had a half-life of 154 min in mouse plasma, and **2**, with a 222-min half-life. Based on potency, selectivity, and in vitro properties, compound **4** (MM3122) and compound **2** (VD2173) were selected as lead candidates and were tested for their in vivo PK in mice. Compounds **5** through **7** were not tested for PK, due their lower antiviral activity and the relatively high Factor Xa activity of **5** and **6**. Shown in Fig. 8, VD2173 (**2**) has a half-life of 2.5 h in mice with a high area under the curve (AUC) and exposure in plasma beyond 24 h. Excitingly, rationally designed TMPRSS2 lead compound MM3122 (**4**) also had excellent PK, with a half-life of 8.6 h in plasma. Both VD2173 and MM3122 were subsequently tested for their lung exposure over a period of 24 h after intraperitoneal (IP) dosing. It was found that both compounds attained high levels of compound in the lung and had excellent AUC but with MM3122 being superior, with a 7.5-h half-life versus 4.2 h for VD2173. The potency of MM3122 versus VD2173 in both the TMPRSS2 enzyme assay and viral assays is magnitudes higher (500-fold), making MM3122 an ideal lead candidate drug suitable for in vivo efficacy studies of COVID-19 and further optimization to be reported in a future communication.

#### Acute 7-d toxicity study of MM3122 (4) in mice.

We further tested lead compound MM3122 for its safety in mice. MM3122 was administered daily to mice at three different single dose levels of 20, 50, and 100 mg/kg via IP injection over a period of 7 d. No adverse effects were observed in any of the treatment groups, and no weight loss or changes to harvested organs (liver, spleen, and kidneys) were noted compared to the control group (*SI Appendix*, Fig. S5).

## Conclusions

TMPRSS2 has been shown to be essential for host cell viral entry and replication of SARS-CoV-2 as discussed above. Based on molecular docking studies using a published homology model of TMPRSS2 (45) and substrate specificity data from PS-SCL, we hypothesized that a set of our existing peptidyl kbt inhibitors of HGFA, matriptase, and hepsin would also inhibit TMPRSS2. Indeed, we demonstrated that these compounds potently inhibit not only TMPRSS2 enzyme activity of recombinant and cell-surface protein, but also host cell entry and toxicity driven by the Spike protein of SARS-CoV-2 into ACE2/TMPRSS2-expressing Calu-3 lung epithelial cells. After further optimization, we identified multiple potent inhibitors of TMPRSS2, with four analogs rationally designed for TMPRSS2 displaying subnanomolar activity both in the enzyme assay and in blocking the entry of VSV-SARS-CoV-2 chimeras into human Clau-3 epithelial lung cells. In addition, several of these compounds displayed excellent potency against MERS, another prominent coronavirus. We further confirmed potent antiviral activity against the wild-type SARS CoV-2 virus with five lead compounds, thus identifying the most promising lead compound MM3122 (**4**). We have clearly established that this TMPRSS2 inhibitor MM3122 is more potent than Remdesivir, Camostat, and Nafamostat. Importantly, this compound also shows no toxicity to Calu-3 cells, in contrast to Remdesivir and Nafamostat, which are toxic at higher concentrations. MM3122 has excellent metabolic stability in both mouse and human plasma as well as outstanding PK and safety in mice. Cyclic peptide VD2173 (**2**) and rationally designed TMPRSS2 inhibitor MM3122 both have an impressive >8-h half-life in mice. In addition to its excellent TMPRSS2 inhibition, PK, and antiviral activity, VD2173 has anticancer efficacy in animal models of lung cancer. Judging from the protease selectivity data and potency of MM3122 and other new compounds, these are predicted to also show corresponding anticancer activity, in particular for HGF-driven prostate cancer for which TMPRSS2 is known to play a key role (30, 51).

Recent studies have identified other TMPRSS family serine proteases that can mediate SARS-CoV-2 entry, including TMPRSS13 (293T-ACE2 and Calu-3 cells) (9, 52, 53) and TMPRSS4 (enteroid cells) (16). Although entry in Calu-3 cells is far less dependent on TMPRSS13 than TMPRSS2 (9, 28), the mechanistic contributions and/or redundancies of these proteases for SARS-CoV-2 entry are not known, nor is their relative inhibition resolved in the scope of this study. Future work in animal models, which can resolve the biological and tissue-specific relevance of protease inhibition, is best poised to answer these questions.

MM3122 represents an advanced lead candidate for clinical development as a novel antiviral drug for COVID-19 and against infections caused by other coronaviruses like MERS in which we showed equipotent activity of MM3122. We are currently optimizing MM3122 and this class of inhibitors for TMPRSS2 selectivity and antiviral potency and, in due course, will test MM3122 and other optimized leads in appropriate animal models of COVID-19. In addition to being novel drugs, selective TMRSS2 inhibitors can be used as valuable chemical probes to help elucidate mechanisms of viral pathogenesis, including host cell−virus interactions, Spike protein processing, and ACE2 receptor binding. Since TMPRSS2 plays a key role as a viral protein processing protease in the pathogenesis of other coronaviruses (SARS-CoV, MERS-CoV) as well as influenza viruses (25, 54–58), MM3122 and this new class of inhibitors may be effective not only against COVID-19 but for infections caused by most or all corona and influenza viruses. Thus, these small-molecule inhibitors of TMPRSS2 not only hold much promise as new drugs to treat SARS-CoV-2 infections but also potentially represent broad-spectrum antivirals.

## Experimental

### TMPRSS2-Protease Domain Expression and Purification.

Periplasm secreted bacterial expression of serine proteases has been widely reported to yield correctly folded high-quality enzyme due to its reducing environment allowing for disulfide formation and fewer proteases being present compared to the cytoplasm (59, 60). We therefore employed a similar strategy for TMPRSS2 protease domain. The human TMPRSS2 extracellular domain (residues 106 through 492) was cloned into a pET28a vector with a *pelB* leader sequence followed by an N-terminal 6x His tag (*SI Appendix*, Fig. S8*A*) using standard Gibson assembly procedures (61). *Escherichia coli* DH5a cells were transformed with the Gibson assembly reaction mixture, and resulting plated colonies were selected and verified with Sanger DNA sequencing for correct assembly of the cloned product. Correctly cloned plasmid was used for transforming *E. coli* BL21(DE3) cells and later used for subsequent expressions. A single colony of BL21(DE3) cells was picked and inoculated into 50 mL of luria broth (LB) with 2% glucose and 50 mg/mL Kanamycin culture and grown overnight at 37 °C, 220 rpm. Liter-scale liquid media (LB 0.1% glucose, 50 mg of Kanamycin) were inoculated with overnight cultures at a starting optical density at a wavelength of 600 nm (OD_600_) of 0.05, and expression was induced with a final concentration of isopropylthio-β-galactoside (IPTG) at 1 mM at OD_600_ of 0.7. Expressions were carried out at 16 °C for ∼72 h. Cells were collected by centrifugation, and the periplasmic extracts were prepared by osmotic shock. Briefly, cell pellets were resuspended in TES buffer [200 mM Tris·HCl pH 8, 0.5 mM (ethylenedinitrilo)tetraacetic acid, 500 mM sucrose] and incubated at 4 °C for 1 h followed by addition of cold water and incubation at 4 °C for 45 min. The periplasmic extract was isolated with centrifugation (10,000 × *g* for 30 min) at 4 °C, and imidazole (10 mM final concentration) and MgCl_2_ (100 mL/L expression culture volume) were added for overnight batch binding with Ni-NTA resin (2 mL of slurry per liter of expression culture volume). Purification was carried out with 10 column volumes of Wash buffer 1 (50 mM Tris, 250 mM NaCl, pH 7.6) and, subsequently, 10 column volumes of wash buffer 2 (50 mM Tris, pH 7.6, 250 mM NaCl, 20 mM imidazole) and eluted with elution buffer (50 mM Tris, pH 7.6, 250 mM NaCl, 4 mM benzamidine, 1 mM CaCl_2,_ 500 mM imidazole) (*SI Appendix*, Fig. S8*C*). SDS/PAGE was performed with fractions of eluate, and those showing the correctly sized band for TMPRSS2 protease domain were pooled. Consolidated fractions were buffer exchanged against 25 mM Citrate pH 6, 250 mM NaCl, 4 mM benzamidine, 1 mM CaCl_2_ to remove excess imidazole and prevent autolysis and concentrated down in the same buffer with 10-kDa NMWL Amicon ultra filtration units. Potential aggregates and degradation products were removed by size exclusion chromatography with 25 mM Citrate pH 6, 250 mM NaCl, 4 mM benzamidine, 1 mM CaCl_2_ and 10% glycerol and Superdex 200 Increase 10/300 GL. TMPRSS2 protease domain containing fractions by SDS/PAGE (*SI Appendix*, Fig. S8*D*) were pooled. A final Western blot against the protease domain (TMPRSS2 monoclonal antibody [M05], clone 2F4) was performed to verify the collected sample to be TMPRSS2 protease domain. The pooled sample was flash frozen for subsequent assays (*SI Appendix*, Fig. S8*E*).

#### Recombinant enzyme inhibition assays.

Recombinant human TMPRSS2 was purchased from Cusabio Technology (CSB-YP023924HU) and assayed in 25 mM Tris·HCl, 150 mM NaCl, 5 mM CaCl_2_, 0.01% Triton X-100, pH 8.0 at a final concentration of 3 nM. To calculate K_M_, Boc-QAR-AMC (Vivitude, MQR-3135-v) was serially diluted in dimethyl sulfoxide (DMSO), and then each diluent was further diluted in assay buffer such that the final substrate concentration in the assay ranged from 0.514 μM to 200 μM with a final DMSO concentration of 0.5%. Assays were performed in a total volume of 30 μL in triplicate wells of black 384-well plates (Nunc 262260), and initial velocity was measured in 35-s intervals for 7 min with excitation of 360 nm and emission of 460 nm on a Biotek HTX plate reader. K_M_ and V_max_ were calculated from Michaelis−Menten plots using GraphPad Prism. For inhibitor studies, compounds were serially diluted threefold in DMSO and then preincubated with TMPRSS2 in assay buffer for 30 min at room temperature. The reaction was initiated following the addition of Boc-QAR-AMC, and fluorescence was measured in 190-s intervals for 90 min. The final concentrations of enzyme and substrate were 3 nM and 86.6 μM, respectively, and the inhibitor concentrations ranged from 2 μM to 0.15 pM. Assays were performed in quadruplicate plates, and IC_50_ was calculated from dose–response curves using GraphPad Prism. IC_50_ values for HGFA, matriptase, hepsin, factor Xa, and thrombin were determined using our published assays (37–39).

### MSP-MS.

Human recombinant TMPRSS2 (100 nM) expressed in yeast (see *Expression of Human Recombinant TMPRSS2 in* Pichia pastoris) was incubated at room temperature with a physiochemically diverse library of 228 tetradecapeptides to a final concentration of 500 nM. Aliquots were removed at different time intervals (15, 60, and 240 min) and subsequently quenched with 1Eq volume of 8 M guanidinium hydrochloride. Samples were desalted using C18 tips (Rainin) and analyzed by liquid chromatography (LC)-MS/MS peptide sequencing using a Quadrupole Orbitrap mass spectrometer (LTQ Orbitrap) coupled to a 10,000-psi nanoACQUITY Ultra Performance Liquid Chromatography System (Waters) for peptide separation by reverse phase LC. Peptides were separated over a Thermo ES901 C18 column (75-μm inner diameter, 50-cm length) coupled to an EASY-SprayTM ion source and eluted by applying a flow rate of 300 nL/min in a 65-min linear gradient from 2 to 50% in Buffer B (acetonitrile, 0.5% formic acid). Survey scans were recorded over a 325 *m/z* to 1,500 *m/z* range, and up to three of the most intense precursor ions (MS1 features of charge ≥ 2) were selected for collision-induced dissociation. Data were acquired using Xcalibur software and processed as previously described (62). Briefly, raw MS data were processed to generate peak lists using MSConvert. Peak lists were then searched in Protein Prospector v.6.2.2 (63) against a database containing the sequences from the 228 tetradecapeptide library. Searches used a mass accuracy tolerance of 20 ppm for precursor ions and 0.8 Da for fragment ions. Variable modifications included N-terminal pyroglutamate conversion from glutamine or glutamate and oxidation of tryptophan, proline, and tyrosine. Searches were subsequently processed using the MSP-xtractor software (https://pharm.ucsf.edu/craik/research/extractor), which extracts the peptide cleavage site and spectral counts of the corresponding cleavage products. Spectral counts were used for the relative quantification of peptide cleavage products.

### Expression of Human Recombinant TMPRSS2 in *Pichia pastoris*.

Human recombinant TMPRSS2 was expressed and purified from yeast following protocols previously described (30), with slight modifications. Briefly, the SRCR and serine protease domain regions of TMPRSS2-N249G were cloned into a pPICZα-B construct (EasySelect Pichia Expression Kit; Invitrogen) according to the manufacturer, transformed into *P. pastoris* strain ×33, and selected on plates containing increasing concentrations of Zeocin. A single colony was grown in 10 mL of buffered medium with glycerol (BMGY) overnight at 30 °C and 230 rpm. The overnight culture was used to inoculate 1 L of BMGY and grown until OD_600_ 2 through 6. Cells were pelleted and resuspended in 100 mL of buffered medium with methanol. Cells were induced with 5% methanol every 24 h for 72 h to 96 h. Secreted TMPRSS2 was precipitated with 70% ammonium sulfate at 4 °C overnight, pelleted at 27,000 × *g* for 45 min, and resuspended in 50 mM Tris pH 8, 0.5 M NaCl, 0.01% CHAPS, and solubilized for 2 h at 4 °C. The solubilized protein was then purified over a gravity column containing soybean trypsin inhibitor immobilized agarose (Pierce). TMPRSS2 was concentrated and then purified with size exclusion chromatography using 50 mM potassium 83 phosphate buffer, pH 6, 150 mM NaCl, 1% glycerol to prevent autoproteolysis.

#### Cell-based TMPRSS2 fluorogenic enzyme inhibition assay.

A PLX304 plasmid–containing human TMPRSS2 open reading frame from the ORFeome Collaboration (Dana-Farber Cancer Institute, Broad Institute of Harvard and Massachusetts Institute of Technology [HsCD00435929]) was obtained from DNASU Plasmid Repository, and a control PLX304 vector was obtained from Addgene. HEK-293T cells were grown in Dulbecco’s Modified Eagle Medium (DMEM) supplemented with 10% fetal bovine serum (FBS) and seeded in a black, 96-well plate (75,000 cells per well). The following day, cells were transfected overnight with either a control plasmid (PLX) or TMPRSS2 (PLX-TMPRSS2) via TransIT LT-1 transfection reagent (Mirus Bio) in 100 μL of OptiMEM per well. Twenty-four hours after transfection, the media was replaced with 80 μL of phosphate-buffered saline (PBS). Inhibitors or PBS alone were added to the wells in the indicated five concentrations and incubated at 25 °C for 15 min. The fluorogenic substrate Boc-QAR-AMC (R&D Biosystems) was then added to each well to a final concentration of 100 μM. Fluorescence (excitation 365 nm, emission 410 nm) was kinetically measured every 15 min for a total time of 150 min at 37 °C using a GloMax plate reader (Promega).

#### Antiviral activity (VSV-SARS-CoV-2 and MERS chimeras).

##### Viruses used.

VSV-eGFP, a recombinant VSV expressing a GFP reporter (depends on the VSV glycoprotein G for entry), has been previously described (64). VSV-SARS-CoV-2, a replication-competent infectious VSV chimera which employs the SARS-CoV-2 Spike (S) protein for viral entry in place of VSV G and expresses eGFP, has also been previously described (41, 65). VSV-MERS was created in the same manner as VSV-SARS-CoV-2, except the MERS Spike missing the terminal 21 amino acids (HCoV-EMC/2012 strain) was inserted in place of VSV G.

##### Experimental procedure.

Human Calu-3 lung epithelial cells or Vero cells (African green monkey kidney) were seeded in a 96-well black plate in DMEM containing 10% FBS for 24 h (37 °C and 5% CO_2_). The next day, cells were pretreated for 2 h with inhibitor or vehicle control (DMSO) in 50 μL of serum-free DMEM, and subsequently infected with VSV-SARS-CoV-2, VSV-MERS, or VSV-eGFP at a multiplicity of infection (MOI) of 0.5. At 7 h postinfection (single round of infection), cells were fixed in 2% formaldehyde, and nuclei was stained with 10 μg/mL Hoechst 33342 (Invitrogen) for 30 min at room temperature. Cells were washed once and stored in PBS after fixation, and automated microscopy was performed using an InCell 2000 Analyzer (GE Healthcare) in the DAPI and fluorescein isothiocyanate (FITC) channels (10× objective, nine fields per well, covering the entire well). Images were analyzed using the Multi Target Analysis Module of the InCell Analyzer 1000 Workstation Software (GE Healthcare) to identify the percentage of GFP-positive cells in each well (top-hat segmentation). The percentage GFP-positive cells for experimental conditions was normalized against the control (DMSO treated) cells and expressed as relative infectivity. GraphPad Prism (version 8.4.2) was used to calculate the EC_50_ of each drug. Statistics (comparison of VSV-SARS-CoV-2 to VSV-eGFP with each drug; Student’s *t* test) were performed in Microsoft Excel. Three biological replicates were performed.

##### Antiviral activity (VSV pseudotype SARS-CoV-2).

We analyzed pseudotype entry driven by the spike protein of SARS-CoV-2 (SARS-2-S) or the glycoprotein of vesicular stomatitis virus (VSV-G) into the TMPRSS2-positive human lung cell line Calu-3 (7, 29). VSV-G was used as a control, as it does not depend on TMPRSS2 for host cell entry. Besides Calu-3 cells, we further used Vero cells (African green monkey, kidney) as a control, as these cells do not express TMPRSS2, and therefore any reduction in SARS-2-S−driven entry would be related to either unspecific side effects or cytotoxicity. Each compound was tested in three separate experiments with independent pseudotype batches. We treated cells (96-well format) with different concentrations of inhibitor or solvent (DMSO) diluted in medium (50 μL per well) for 2 h at 37 °C and 5% CO_2_, then we added 50 μL of pseudotype on top and incubated for 16 h at 37 °C and 5% CO_2_. Next, we measured virus-encoded firefly luciferase activity in cell lysates (indicator of pseudotype entry into target cells). The data were normalized against control (DMSO-treated cells = 100% pseudotype entry) and plotted with GraphPad Prism (version 8.3.0), to calculate effective concentration EC_50_ and perform statistics (comparison against the respective control; two-way ANOVA with Dunnett’s posttest).

#### Antiviral activity (wild-type SARS-CoV-2).

We seeded Calu-3 cells onto a white, flat bottom 96-well plate at 10,000 cells per well in DMEM supplemented with 10% heat-inactivated FBS, 100 U/mL Penicillin-Streptomycin (Life Technologies, Inc., 15140-163), and buffered with 10 mM Hepes and incubated in a humidified 5% CO_2_ incubator at 37 °C. Sixteen hours later, the media was removed and replaced with the 50 μL per well of the same media but containing 2% heat-inactivated FBS (D-2) instead of 10%. In D-2, compounds were serially diluted threefold in a nine-point series and added in a 25-μL volume to the 96-well plate to make final concentrations ranging from 50 μM to 7.6 nM on the assay plate for 1 h. We then infected the cells (with compounds still on them) with 25 μL per well of SARS-CoV-2 at an MOI of 0.1 plaque forming units (PFU) per cell, with a final total volume of 100 μL per well. We incubated the plates for 72 h as described above and then added 25 μL per well of CellTiter-Glo Reagent (prepared as directed by manufacturer; Promega, G7573), followed by 5 min of shaking and 20 min of room temperature incubation before detecting luminescence with a Biotek Synergy H1 plate reader. Compound cytotoxicity was determined using the same assay, but, instead of adding virus, we added 25 μL of D-2. These experiments were performed three times, and we performed nonlinear regression on log(inhibitor) vs. response with variable slope and four parameters on compiled results from the three experiments. Likewise, IC_50_s were calculated based on all three biological replicates.

### Animal Studies.

All animal procedures were performed following the guidelines and approval of the Washington University’s Institutional Animal Care and Use Committee. Animals were maintained in a controlled temperature (22 °C ± 0.5 °C) and lighting (12 L:12 D) environment. Standard laboratory chow and water were supplied ad libitum.

### Mouse Toxicity Studies.

NOD-scid IL2Rgnull (NSG) male and female mice (three per group) were injected with 0, 20, 50, or 100 mg/kg MM3122 (in 1 to 5% DMSO/95% saline; IP) daily for 7 d. Animal health was assessed daily for drug toxicity (ruffled fur, discharge from the eyes, weight loss, dehydration, lethargy, hypothermia, abnormal tissue growth), and body weights were measured every 2 d to 3 d. At the end of the experiment, animals were killed, and organs were inspected. Liver, spleen, and both kidneys were collected and weighed.

### Mouse PK Studies.

MM3122 was tested for its PK in mice (single IP dosing, actual dose 16.7 mg/kg). Eight animal groups contained three animals each (male, CD-1), with plasma drawn and lung tissue harvested at eight different sampling time points (0.08, 0.25, 0.5, 1, 2, 4, 8, and 24 h) post dosing. VD2173 was tested for its PK in mice (single IP dosing, actual dose 16.7 mg/kg). Three animal groups contained three animals each (male, CD-1), with plasma drawn and lung tissue harvested from each group of animals at three different sampling time points (0.25, 8, and 24 h) post dosing. Bioanalysis of plasma and lung tissue extracts were performed using standard LC/MS/MS techniques.

### Supplementary Data.

Single point inhibition data using recombinant protease domain with FRET substrate for **4** through **6** are provided in *SI Appendix*, Fig. S1. Protease selectivity data for **1** (ZFH7116) and **2** (VD2173) are provided in *SI Appendix*, Tables S1 and S2. Synthesis and NMR and high-performance liquid chromatography mass spectrometry (HPLC-MS) spectra of new compounds **2**, **4** through** 7**, and **19** through **21** are provided in *SI Appendix*, Schemes S1−S3. K_m_ curve for Boc-QAR-AMC using full-length TMPRSS2 is provided in *SI Appendix*, Fig. S2. IC_50_ inhibition curves of full-length TMPRSS2/Boc-QAR-AMC are provided in *SI Appendix*, Fig. S3. Cell-based enzyme activity of compounds **1** and **2** in HEK-293 cells is provided in *SI Appendix*, Fig. S4. Acute toxicity of MM3122 (**4**) is provided in *SI Appendix*, Fig. S5. Activity of **1** and **2** and Camostat using Vero cells in pseudotype is provided in *SI Appendix*, Fig. S6 and in chimeric VSV-SARS-CoV-2 viruses in *SI Appendix*, Fig. S7. Expression and purification of the TMPRSS2 protease domain are provided in *SI Appendix*, Fig. S8.

## Supplementary Material

Supplementary File

## Data Availability

All study data are included in the article and *SI Appendix*.
